# Supplementary material for: On the Quina side: A Neanderthal bone industry at Chez-Pinaud site, France
Source: PLoS One. 2023 Jun 14;18(6):e0284081. doi: 10.1371/journal.pone.0284081 (PMC10266661; doi:10.1371/journal.pone.0284081)

S1 Fig. Archaeological deposits of the Chez-Pinaud site (Jonzac, France). (a) location of the excavation area in 2019-2021. (b) left stratigraphic cut (after Airvaux and Soressi 2005). (c) view of the “bone-bed”, 2021 excavation (photos: W. Rendu).

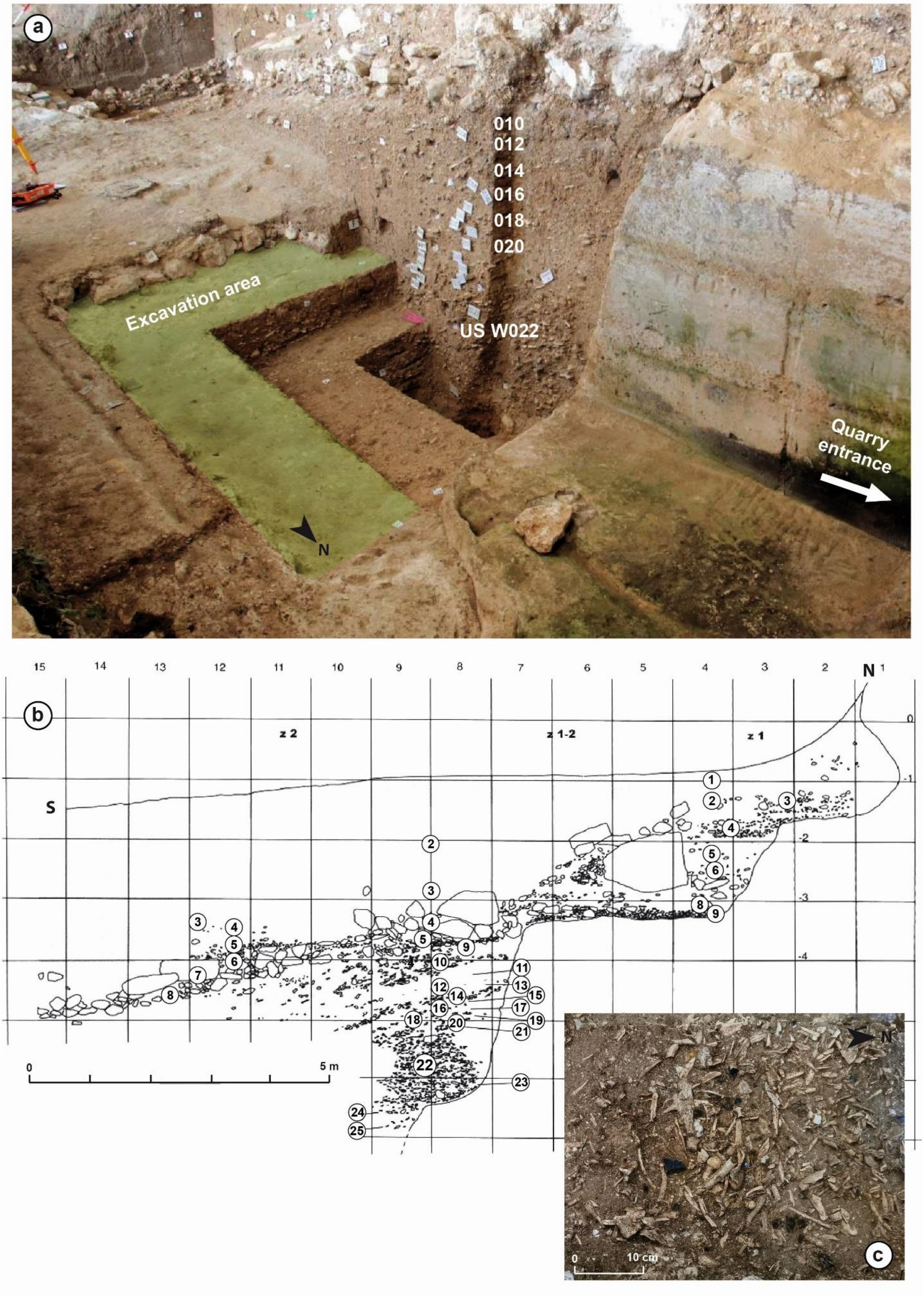

Supplement: S1 Fig — a) location of the excavation area in 2019–2021; b) left stratigraphic cut (after Airvaux and Soressi 2005); c) view of the “bone-bed”, 2021 excavation (photos: W. Rendu). (PDF) [file pone.0284081.s001.pdf]
