# Supplementary material for: On the Quina side: A Neanderthal bone industry at Chez-Pinaud site, France
Source: PLoS One. 2023 Jun 14;18(6):e0284081. doi: 10.1371/journal.pone.0284081 (PMC10266661; doi:10.1371/journal.pone.0284081)

S2 Fig. Experiments with replicas of Mousterian type bone tools. (a) fracturation by direct percussion on anvil; (b) fractured fresh long bones of Bovinae. (c) regular flakes from bone fracturing (d) impact of direct percussion. (e) retouched bone blank. (f) flake from retouch. (g) Mousterian side scrapers shaping. (h) meat cutting (i) hair removing from skin. (j) wooden peeling. (k) plant harvesting. (l) soil digging (photos: H. Plisson).

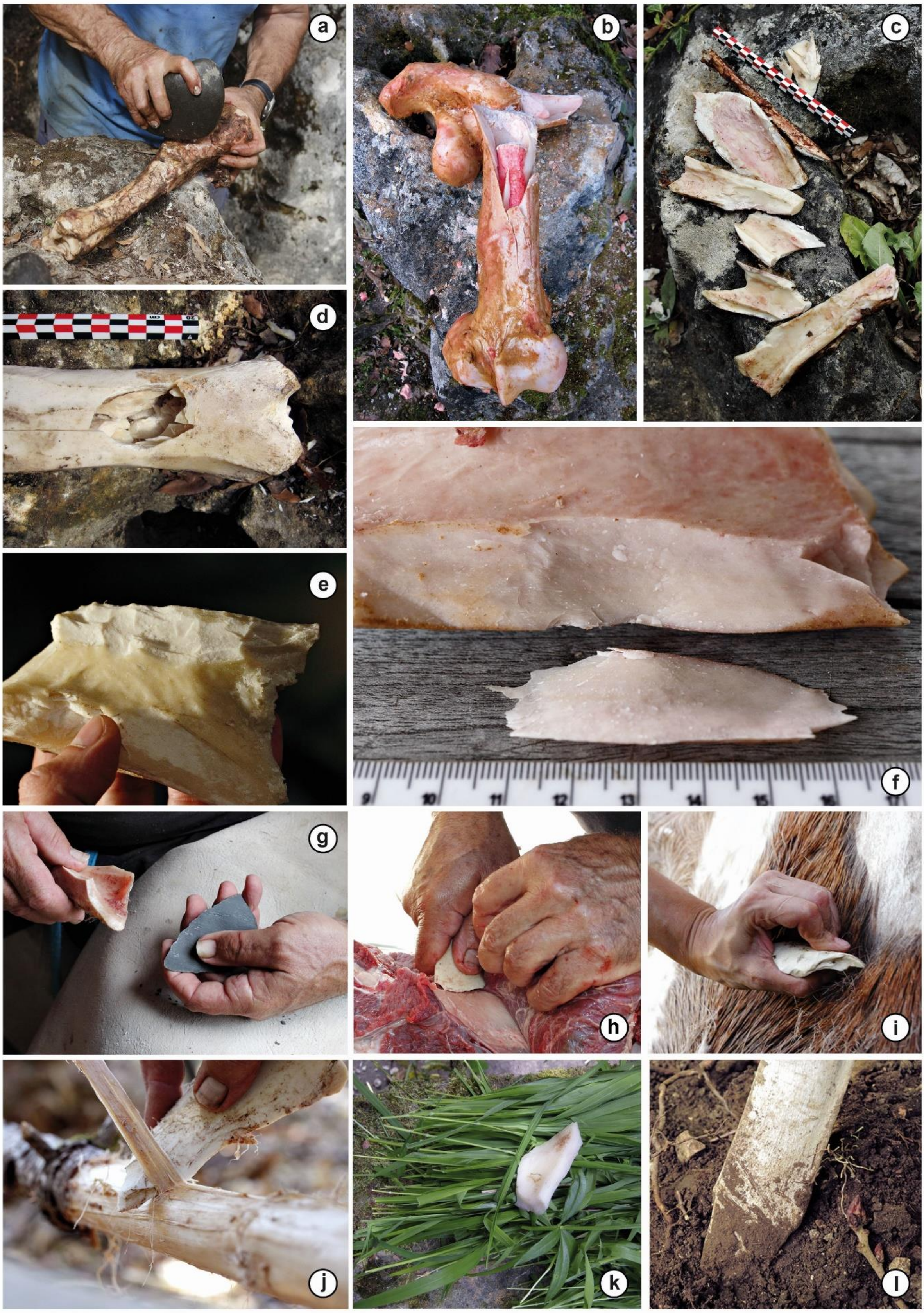

Supplement: S2 Fig — a) fracturation by direct percussion on anvil; b) fractured fresh long bones of Bovinae; c) regular flakes from bone fracturing; d) impact of direct percussion; e) retouched bone blank; f) flake from retouch; g) Mousterian side scrapers shaping; h) meat cutting; i) hair removing from skin; j) wooden peeling; k) plant harvesting; l) soil digging (photos: H. Plisson). (PDF) [file pone.0284081.s002.pdf]
