# Supplementary material for: On the Quina side: A Neanderthal bone industry at Chez-Pinaud site, France
Source: PLoS One. 2023 Jun 14;18(6):e0284081. doi: 10.1371/journal.pone.0284081 (PMC10266661; doi:10.1371/journal.pone.0284081)
Supplement: S3 Fig — a, c–d) Abri Peyrony; b) Pech de l’Azé (after Soressi et al. 2013); e) Zaskalnaya VI (after Stepanchuk et al. 2017); f) Chagyrskaya Cave (after Baumann et al. 2020); g) Abri des Canalettes (after Patou-Mathis 1993); h) Axlor (after Mozota Holgueras 2012); i–k) La Quina (after Henri-Martin 1907–1910); l–m) Grotte du Noisetier (after Oulad El kaïd 2016). (PDF) [file pone.0284081.s003.pdf]

S3 Fig. Examples of ribs with smoothed end discovered in other Middle Paleolithic contexts. (a), (c–d) Abri Peyrony. (b) Pech de l'Azé (after Soressi et al. 2013). (e) Zaskalnaya VI (after Stepanchuk et al. 2017). (f) Chagyrskaya Cave (after Baumann et al. 2020). (g) Abri des Canalettes (after Patou-Mathis 1993). (h) Axlor (after Mozota Holgueras 2012). (i–k) La Quina (after Henri-Martin 1907–1910). (l–m) Grotte du Noisetier (after Oulad El kaïd 2016).

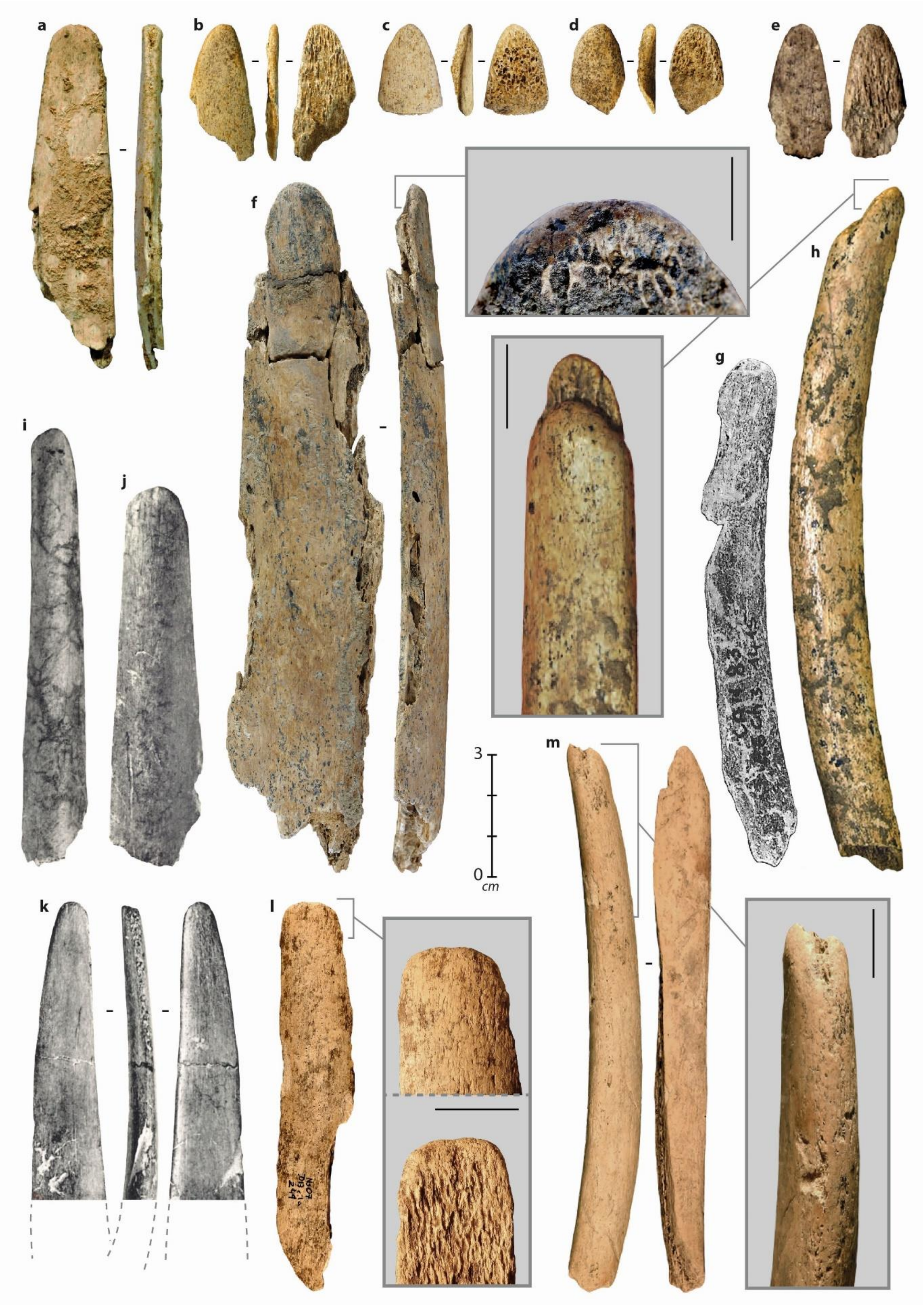

References

- Baumann, M., Plisson, H., Rendu, W., Maury, S., Kolobova, K. and Krivoshapkin, A. (2020). The Neandertal bone industry at Chagyrskaya cave, Altai Region, Russia. Quaternary International 559: 68–88.
- Henri-Martin, L. (1907-1910). Recherches sur l’Evolution du Moustérien dans le Gisement de la Quina (Charente), Premier Volume 1, Industrie Osseuse. Paris: Schleicher Frères.
- Mozota Holgueras, M.M. (2012). El hueso como materia prima: El utillaje óseo del final del Musteriense en el sector central del norte de la Península Ibérica. Tesis Doctoral, Universidad de Cantabria.
- Oulad El Kaïd, C. (2016). L’exploitation des matières osseuses au Paléolithique inférieur et moyen : l’exemple de la grotte du Noisetier (Fréchet-Aure, Hautes-Pyrénées). Master II, Université de Toulouse Jean-Jaurès.
- Patou-Mathis, M. (1993). Etude taphonomique et palethnographique de la faune de l'Abri des Canalettes. In : Meignen, L. (ed), L'Abri des Canalettes. Paris: CNRS, pp.199–237.
- Soressi, M., McPherron, S.P., Lenoir, M., Dogandžić, T., Goldberg, P., Jacobs, Z., Maigrot, Y., Martisius, N.C., Miller, C.E., Rendu, W., Richards, M., Skinner, M.M., Steeles, T.E., Talamo, S. and Texier, J.P. (2013). Neandertals made the first specialized bone tools in Europe. PNAS 110: 14186–14190.
- Stepanchuk, V.N., Vasilyev, S.V., Khaldeeva, N.I., Kharlamova, N.V., Borutskaya, S.B. (2017). The last Neanderthals of Eastern Europe: Micoquian layers IIIa and III of the site of Zaskalnaya VI (Kolosovskaya), anthropological records and context. Quaternary International 428: 132–150.
