# Supplementary material for: On the Quina side: A Neanderthal bone industry at Chez-Pinaud site, France
Source: PLoS One. 2023 Jun 14;18(6):e0284081. doi: 10.1371/journal.pone.0284081 (PMC10266661; doi:10.1371/journal.pone.0284081)
Supplement: S4 Fig — a) Castel di Guido (after Villa et al. 2021); b) Nova de Columbeira (after Zilhão et al. 2011); c) Vauffrey (after Vincent 1993); d) Poggeti Vecchi (after Aranguren et al. 2019); e) Bois-Roche (after Vincent 1993); f) Combe-Grenal (after Tartar and Costamagno 2016); g–h) Chagyrskaya (after Baumann et al. 2020); i) Gran Dolina (after Rossel et al. 2011); j) Abric Romaní (after Carbonel et al. 1994). (PDF) [file pone.0284081.s004.pdf]

S4 Fig. Examples of retouched bone artifacts discovered in pre-AMH contexts. (a) Castel di Guido (after Villa et al. 2021). (b) Nova de Columbeira (after Zilhão et al. 2011). (c) Vauffrey (after Vincent 1993). (d) Poggeti Vecchi (after Aranguren et al. 2019). (e) Bois-Roche (after Vincent 1993). (f) Combe-Grenal (after Tartar and Costamagno 2016). (g–h) Chagyrskaya (photo: M. Baumann). (i) Gran Dolina (after Rossel et al. 2011). (j) Abric Romaní (after Carbonel et al. 1994).

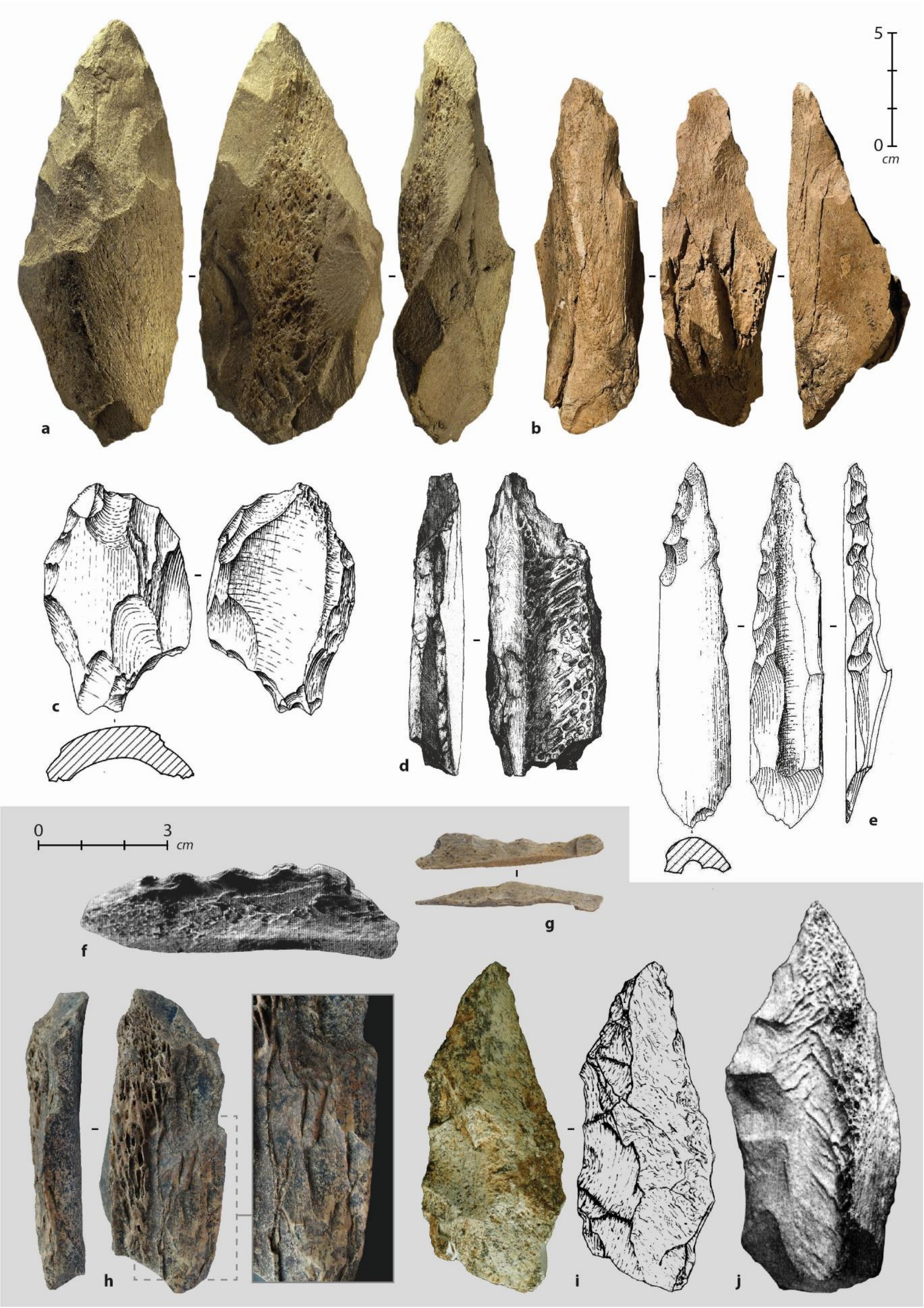

References

- Aranguren, B., Grimaldi, S., Benvenuti, M., Capalbo, C., Cavanna, F., Cavulli, F., et al. (2019). Poggetti Vecchi (Tuscany, Italy): A late Middle Pleistocene case of human-elephant interaction. Journal of Hulan Evolution 113: 32-60.
- Carbonel, E., Giralt, S., Vaquero, M. (1994). Abric Romani (Capellades, Barcelone, Espagne) : une importante séquence anthropisée au Pléistocène superieur. Bulletin de la Société préhistorique française 91: 47-55.
- Rosell, J., Blasco, R., Campeny, G., Díez, J.C., Alcalde, A.R., Menéndez, L., et al. (2011). Bone as technological raw material at the Gran Dolina site (Sierra de Atapuerca, Burgos, Spain). Journal of Human Evolution 6: 125-131.
- Tartar, É., Costamagno, S. (2016). L'utilisation des matières osseuses au Moustérien. In: Turq, A., Faivre, J.-Ph., Maureille, B., Lahaye, Ch., Bayle, P. (eds), Néandertal à la loupe. Les Eyzies: Musée National de Préhistoire, pp. 89-97.
- Villa, P. and d'Errico, F. (2001). Bone and ivory points in the Lower and Middle Paleolithic of Europe. Journal of Human Evolution 41: 69–112.
- Vincent, A. (1993). L'outillage osseux au Paléolithique moyen : une nouvelle approche. Thèse de Doctorat, Université Paris 10.
- Zilhão, J., Cardoso, J.L., Pike, A.W.G., Weninger, B. (2011). Gruta Nova da Columbeira (Bombarral, Portugal): Site stratigraphy, age of the Mousterian sequence, and implications for the timing of Neanderthal extinction in Iberia. Quatär 58: 93-112.
