# Supplementary material for: On the Quina side: A Neanderthal bone industry at Chez-Pinaud site, France
Source: PLoS One. 2023 Jun 14;18(6):e0284081. doi: 10.1371/journal.pone.0284081 (PMC10266661; doi:10.1371/journal.pone.0284081)
Supplement: S1 Table — (PDF) [file pone.0284081.s005.pdf]

S1 Table. Microtomographic recording parameters of the experimental and archaeological tools from Chez-Pinaud site.

	Identification number	Raw material	Tool type	Scanned area	Resolution (µm)	Slice nb.	Correction value	kV	µA
Archaeological tools	CPN19-5	Diaphysis large size ungulate	Retouched	Complete	26	1625	10.40	120	120
	CPN19-529	Diaphysis medium size ungulate	Retouched	Complete	26	1625	10.40	120	120
	CPN19-534	Humerus large size ungulate	Beveled	Complete	35	2125	8.32	120	120
	CPN19-888	Diaphysis large size ungulate	Beveled Retouched	Complete	35	1500	9.13	120	120
	CPN19-1014	Horse humerus	Beveled Retouched Retoucher	Complete	67	1200	2.46	120	250
				ROI* 1	21	2550	3.52	120	250
	CPN19-2020	Horse tibia	Retoucher	Complete	62	1200	2.48	120	250
				ROI 1	22	2250	4.49	120	250
	CPN19-2132	Diaphysis large size ungulate	Beveled ? Retouched Retoucher	Complete	43	1375	7.12	120	120
	CPN20-3581	Rib large size ungulate	Smoothed	Complete	54	1275	8.31	120	100
				ROI 1	14	1625	27.10	120	100
				ROI 2	5	2550	74.83	120	120
	CPN20-3609	Bison metatarsal	Beveled Smoothed	Complete	43	1375	8.41	120	120
Experimental tools	Exp-10	Cow tibia	Beveled	Complete	89	1200	2.82	120	250
				ROI 1	13	2250	8.24	120	250
				Zone 2	13	2250	1.69	120	250
	Exp-13	Red deer femur	Beveled Retouched	Complete	89	1200	2.82	120	250
				ROI 1	24	2250	5.22	120	250
				ROI 2	24	2250	2.12	120	250
	Exp-46	Cow tibia	Retoucher Retouched	Compete	98	1500	2.78	120	250
				ROI 1	21	2250	6.18	120	250
	Exp-57	Cow tibia	Retoucher	Complete	98	1200	3.18	120	250
				ROI 1	21	2550	3.22	120	250
	Exp-58	Cow tibia	Retoucher	Complete	98	1200	3.18	120	250
				ROI 1	33	2250	4.02	120	250

*ROI = Region of interest
