# Supplementary material for: On the Quina side: A Neanderthal bone industry at Chez-Pinaud site, France
Source: PLoS One. 2023 Jun 14;18(6):e0284081. doi: 10.1371/journal.pone.0284081 (PMC10266661; doi:10.1371/journal.pone.0284081)
Supplement: S2 Table — (PDF) [file pone.0284081.s006.pdf]

S2 Table. Experimental samples analyzed in µCT.

	Exp-10	Exp-13	Exp-46	Exp-57	Exp-58
Raw material	Cow Tibia	Red deer Femur	Cow Tibia	Cow Tibia	Cow Tibia
Time after death (months)	4	10	0	1	15
1st fracturing step	Direct percussion/ on anvil	Direct percussion/ on anvil	Direct percussion/ on anvil	Direct percussion/ on anvil	Direct percussion/ on anvil
Shaping	None	Retouched	Retouched	None	None
Shaping technique	None	Direct percussion/ Soft hammer	Direct percussion/ Soft hammer	None	None
Tool type	Beveled	Beveled	Retoucher	Retoucher	Retoucher
Length (cm)	15.9	13.21	16.84	11.47	18.1
Width (cm)	3.27	4.41	5.97	3.61	4.96
Thickness (cm)	1.24	0.71	1.29	1.54	1.23
Worked material	Fresh *Corylus avellana*	Fresh *Corylus avellana*	“Bergerac” flint	“Bergerac” flint	“Bergerac” flint
Activity	Handle manufacture	Handle manufacture	Scraper shaping	Scraper shaping	Scraper shaping
Task	Branch hollowing	Branch hollowing	Edge retouching	Edge retouching	Edge retouching
Time (mn.)	20	20	< 5	< 5	< 5
