# Supplementary material for: On the Quina side: A Neanderthal bone industry at Chez-Pinaud site, France
Source: PLoS One. 2023 Jun 14;18(6):e0284081. doi: 10.1371/journal.pone.0284081 (PMC10266661; doi:10.1371/journal.pone.0284081)
Supplement: S3 Table — (PDF) [file pone.0284081.s007.pdf]

S3 Table. Reports (non-exhaustive) of smoothed-end ribs from Neanderthal contexts in Eurasia.

Loc	Site	Nb.	Industry	Dates	Ref.
France	Abri Peyrony	3	MTA	MIS 3 (43 – 37 ky BP)	Soressi et al. 2013
	Noisetier	2	Discoid	MIS 3 (42 ky BP)	Oulad El Kaïd 2016
	La Quina	3	MAT/Denticulate	MIS 3 (48 - 40 ky BP)	Henri-Martin 1907-1910<br>Debénath et al. 1998
	Lartet	4	Levallois	MIS 3 (48 - 35 ky BP)	Debénath and Duport 1971
	Pech de l'Azé	1	MTA	MIS 3 (51 ky BP)	Soressi et al. 2013
	Pradayrol	1	Discoid<br>Levallois	MIS 3	Villeneuve et al. 2019
	Canalettes	1	Levallois	MIS 5-4 (73 ky BP)	Patou-Mathis 1993<br>Valladas et al. 1987
	Vaufrey	1	Mousterian	MIS 7-6 (270-142 ky BP)	Vincent 1993
Spain	Cueva Mórin	1	MTA (Vasconian)	MIS 3 (>43 ky BP)	Freeman 1971<br>Maíllo-Fernández et al. 2014
	Axlor	1	Quina	MIS 3 (>47 ky BP)	Mozota Holgueras 2012<br>Gómez-Olivencia et al. 2018
Germany	Salzgitter-Lebenstedt	8	Levallois	MIS 3 (55 – 48 ky BP)	Gaudzinski 1999<br>Pastoors 2009
Crimea	Zaskalnaya VI	1	Levallois (Ak-Kaya)	MIS 3 (39 – 30 ky BP)	Stepanchuk et al. 2017
Siberia	Chagyrskaya	2	Micoquian (Sibiryachika)	MIS 4-3 (60 – 50 ky BP)	Baumann et al. 2020

References

- Baumann, M., Plisson, H., Rendu, W., Maury, S., Kolobova, K. and Krivoshapkin, A. (2020). The Neandertal bone industry at Chagyrskaya cave, Altai Region, Russia. Quaternary International 559: 68–88.
- Debénath, A., Duport, L. (1971). Os travaillés et os utilisés de quelques gisements préhistoriques Charentais (Paléolithique ancien et moyen). B Soc Archéol Hist Charente 1971 :189–202.
- Debénath, A., Jelinek, A.J., Armand, D., Chase, P.G., Dibble, H.L., Mercier, N., Renault-Miskovsky, J., Tillier, A.-M., Valladas, H., Vandermeersch, B. (1998). Nouvelles fouilles à La Quina (Charente) : résultats préliminaires. Gallia Préhistoire 40:29–74.
- Freeman, L.G. (1971). El hueso trabajado musteriensede Cueva Morin. In: Gonzalez Echegaray, J. (ed.), Cueva Morin Excavaciones 1966-1968. Patronatode las Cuevas Prehistoricas, Santander, pp 135-61.
- Gaudzinski, S. (1999). Middle Palaeolithic bone tools from the open-air site Salzgitter-Lebenstedt (Germany). Journal of Archaeological Science 26:125–141.
- Gómez-Olivencia, A., Sala, N., Núñez-Lahuerta, C., Sanchis, A., Arlegi, M., Rios-Garaizar, J. (2018). First data of Neandertal bird and carnivore exploitation in the Cantabrian Region (Axlor; Barandiaran excavations; Dima, Biscay, Northern Iberian Peninsula). Sci Rep 8:10551
- Henri-Martin, L. (1907-1910). Recherches sur l'Evolution du Moustérien dans le Gisement de la Quina (Charente), Premier Volume 1, Industrie Osseuse. Paris: Schleicher Frères.
- Maíllo-Fernández, J.M., Arteaga, C., Iriarte-Chiapusso, M.-J., Fernández, A., Wood, R., Bernaldo de Quirós, F. (2014). Cueva Morín (Villanueva de Villaescusa, Cantabria). In: Sala Ramos, R. (ed.), Pleistocene and Holocene Hunter-Gatherers in Iberia and the Gibraltar Strait: The Current Archaeological Record. Universidad de Burgos, Burgos, pp 72–-78.
- Mozota Holgueras, M.M. (2012). El hueso como materia prima: El utillaje óseo del final del Musteriense en el sector central del norte de la Península Ibérica. Tesis Doctoral, Universidad de Cantabria.
- Oulad El Kaïd, C. (2016). L'exploitation des matières osseuses au Paléolithique inférieur et moyen : l'exemple de la grotte du Noisetier (Fréchet-Aure, Hautes-Pyrénées). Master II, Université de Toulouse Jean-Jaurès.
- Pastoors, A. (2009). Blades? Thanks, no interest! Neanderthals in Salzgitter-Lebenstedt. Quartär 56:105–118.
- Patou-Mathis, M. (1993). Etude taphonomique et palethnographique de la faune de l'Abri des Canalettes. In : Meignen, L. (ed), L'Abri des Canalettes. Paris: CNRS, pp.199–237.
- Soressi, M., McPherron, S.P., Lenoir, M., Dogandžić, T., Goldberg, P., Jacobs, Z., Maigrot, Y., Martisius, N.C., Miller, C.E., Rendu, W., Richards, M., Skinner, M.M., Steeles, T.E., Talamo, S. and Texier, J.P. (2013). Neandertals made the first specialized bone tools in Europe. PNAS 110: 14186–14190.
- Valladas, H., Chadelle, J.-P., Geneste, J.-M., Joron, J.-L., Meignen, L., Texier P.-J. (1987). Datations par la thermoluminescence de gisement moustériens du sud de la France. Anthropologie 91: 211–226.
- Stepanchuk, V.N., Vasilyev, S.V., Khaldeeva, N.I., Kharlamova, N.V., Borutskaya, S.B. (2017). The last Neanderthals of Eastern Europe: Micoquian layers IIIa and III of the site of Zaskalnaya VI (Kolosovskaya), anthropological records and context. Quaternary International 428: 132–150.

- Villeneuve, Q., Faivre, J.-P., Turq, A., Guadelli, J.-L. (2019). Étude techno-économique du Moustérien de Pradayrol (Caniac-du-Causse, Lot) : Entre mobilité des ressources lithiques et adaptation aux matériaux locaux, un exemple de gestion complémentaire des quartzs et silex au Paléolithique moyen récent en Quercy. Comptes Rendu Palevol 18:251–267.
- Vincent, A. (1993). L'outillage osseux au Paléolithique moyen : une nouvelle approche. Thèse de Doctorat, Université Paris 10.
