# Supplementary material for: On the Quina side: A Neanderthal bone industry at Chez-Pinaud site, France
Source: PLoS One. 2023 Jun 14;18(6):e0284081. doi: 10.1371/journal.pone.0284081 (PMC10266661; doi:10.1371/journal.pone.0284081)
Supplement: S4 Table — (PDF) [file pone.0284081.s008.pdf]

S4 Table. Reports (non-exhaustive) of knapped bone tools from pre-AMH contexts in Eurasia.

Loc	Site	Nb.	Industry	Dates	Ref.
France	Combe-Grenal	1<br>1	Mousterian Levallois<br>Denticulate	MIS 3 (39-38 ky BP)	Bordes 1961<br>Vincent 1993<br>Tartar and Cotamagno 2016
	Jonzac	7	Quina	MIS 4 (72 ky BP)	Rendu et al. 2020<br>Richter et al. 2013
	Vaufrey	1	Mousterian Levallois	MIS 4 (74 ky BP)	Vincent 1993<br>Tartar and Costamagno 2016
	Pié-Lombard	4	Levallois	MIS 5 (70 ky BP)	Texier 1974<br>Texier et al. 2011
	Rigabe	6	Levallois	MIS 3-5	Defleur 1988<br>Brugal et al. 2020
	La Ferrassie	1	Mousterian	NR	Bordes, 1961
	Bois-Roche	+/-15	Mousterian	NR	Vincent 1993
	Baume de Gigny	1	Mousterian	NR	Vuillemey 1989
Germany	Rhede	1	Micoquian (Keilmesser)	MIS 5 (70 ky BP)	Tromnau 1983<br>Baales and Stapel 2015
	Sirgenstein	1	Mousterian	NR	Hahn 1976<br>Ono 2006
Belgium	Trou Magrite	1	Mousterian	MIS 3	Personal inventory<br>Jimenez et al. 2016
Italy	Fumane	1	Levallois	MIS 3 (42 ky BP)	Romandini et al. 2014<br>Peresani et al. 2013
	Poggeti Vecchi	10	Mousterian	MIS 6-7 (171 ky BP)	Aranguren et al. 2019
	Casal de'Pazzi	1	Protopontinian	MIS 7 (270-250 ky BP)	Anzidei and Gioia 1992<br>Marra et al. 2018<br>Villa et al. 2021
	La Polledrara	+/-3	Acheulean (without bifaces)	MIS 9 (324 ky BP)	Santucci et al. 2016<br>Villa et al. 2021
	Lademagne	+/-2	Acheulean	MIS 10-11 (405-389 ky BP)	Pereira et al. 2018<br>Villa et al. 2021
	Castel di Guido	81	Acheulean	MIS 11 (395 ky BP)	Boschian and Saccà 2015<br>Villa et al. 2021

	Fontana Ranuccio	5	Acheulean	MIS 11 (407 ky BP)	Biddittu and Serge 1982<br>Pereira et al. 2018<br>Villa et al. 2021
	Malagrotta	+/-6	Acheulean (without bifaces)	MIS 11 (451-378 ky BP)	Marra and Gatta 2019<br>Villa et al. 2021
	Pontecorvos	1	Acheulean	NR	Biddittu and Serge 1982<br>Villa et al. 2021
Spain	Axlor	6	Quina	MIS 3 (>47 ky BP)	Mozota Holgueras 2012<br>Baldéon 1999
	Peña Miel	+/- 22	Quina	MIS 3 (+/-50 ky BP)	Barandiarán 1987<br>Montes et al. 2001
	Abric Romaní	1	Denticulate	MIS 4-3 (61-39 ky BP)	Tartar and Costamagno 2016<br>Carbonel et al. 1994
	Gran Dolina	2	Mousterian	MIS 9 (372-244 ky BP)	Rossel et al. 2011
	Bolomor	1	Denticulate	MIS 9 (350 ky BP)	Rossel et al. 2015
Portugal	Nova de Columbeira	1	Mousterian	(87 ky BP)	Zilhão et al. 2011
Czech Republic	Kůlna	42	Micoquian	MIS 3 (125-45 ky BP)	Vincent 1993
		27	Taubachian		
		4	Levallois		
Russia	Denisova	4	Levallois (C 11.4)	MIS 5 (105 ky BP)	Jacobs et al. 2019<br>Kozlikin et al. 2020
	Chagyrskaya	49	Micoquian (Sibiryachika)	MIS 4-3 (60 – 50 ky BP)	Baumann et al. 2020
Japan	Tategahana	> 3	NR	MIS 3	Ono 2006<br>Nojiri group 1985
	Jinniushan Loc A	NR	NR	(230-200 ky BP)	Ono 2006

References

- Anzidei, A.P., Gioia, P. (1992). The lithic industry at Ribibbia, Casal de'Pazzi. In: Papers of the fourth conference of Italian archaeology, pp. 155-179.
- Aranguren, B., Grimaldi, S., Benvenuti, M., Capalbo, C., Cavanna, F., Cavulli, F., et al. (2019). Poggetti Vecchi (Tuscany, Italy): A late Middle Pleistocene case of human-elephant interaction. Journal of Hulan Evolution 113: 32-60.
- Baales, M., Stapel, B. (2015). Ein Faustkeil aus Knochen: ein seltenes Werkzeug des Neandertalers aus Westfalen. Archäologie 2015: 244–245.
- Baldeón, A. (1993). El yacimiento de Lezetxiki (Gipuzkoa, País Vasco), Los niveles musterienses. Munibe 45: 3–97.
- Barandiarán, I. (1987). Manipulación y uso de restos óseos". En P. Utrilla et al. (ed.), La cueva de Peña Miel, Nieva de Cameros, La Rioja. Excavaciones Arqueológicas en España 154: 87-101.
- Baumann, M., Plisson, H., Rendu, W., Maury, S., Kolobova, K. and Krivoshapkin, A. (2020). The Neandertal bone industry at Chagyrskaya cave, Altai Region, Russia. Quaternary International 559: 68–88.
- Biddittu, I., Serge, A.G. (1982). Utilizzazione dell'osso nel Paleolitico inferiore italiano. In: Atti della XXIII ReunioneScientifico dell' Istituto italiano di Preistoria e Protostorianel Lazio, actes du colloque international (Florence, 7-9 mai1980). Florence: Istituto Italiano di Preistoria e Protostoria, pp. 89-105.
- Bordes, F. (1961). Typologie du Paléolithique Ancient et Moyen. Bordeaux: Imprimerie Delmas.
- Boschian, G., Saccà, D. (2015). In the elephant, everything is good: Carcass use and re-use at Castel di Guido (Italy). Quaternary International 361: 288–296.
- Brugal, J.-P., Argant, A., Boudadi-Maligne, M., Crégut-Bonnour, E., Croitor, R., Fernandez, P., Fourel, J.-B., Fosse, P., Guadelli, J.-L., Labe, B. et al. (2020). Pleistocene herbiores and carniores from France: An update overiew of the literature, sites and taxonomy. Annales de Paléontologie 106, pp.102384.
- Carbonel, E., Giralt, S., Vaquero, M. (1994). Abric Romani (Capellades, Barcelone, Espagne) : une importante séquence anthropisée au Pléistocène superieur. Bulletin de la Société préhistorique française 91: 47-55.
- Defleur, A., (1988). Contribution à la connaissance de l'industrie osseuse du Paléolithique moyen. Bulletin de la Société préhistorique française 5 : 138–140.
- Hahn, J. (1976). Der Sirgenstein, eine urgeschichtliche Höhlenstation im Achtal. Kulturdenkmale in Baden-Württemberg. Blatt 24: 4.
- Jimenez, E.L., Smolderen, A., Jadin, I., Germonpré, M. (2016). Exhumation de la collection faunique d'Edouart Dupont provenant du Trou Magrite (Pont-à-Lesse), Quelles données et quelles perspectives pour une collection du XIXe siècle ? Notae Praehistoricae 36: 167–190.
- Jacobs, Z., Li, B., Shunkov, M.V., Kozlikin, M.B., Bolikhovskaya, N.S., Agadjanian, A.K., Uliyanov, V.A., Vasiliev, S.K., O'Gorman, K., Derevianko, A.P., Roberts, R.G. (2019). Timing of archaic hominin occupation of Denisova Cave in southern Siberia. Nature 565: 594–599.
- Kozlikin, M., Rendu, W., Maury, S., Plisson, H., Baumann, M., Shunkov, M. (2020). Unshaped bone tools from Denisova Cave, Altai. Archaeol Ethnol Anthropol Eurasia 48: 16–28.
- Marra, F., Gatta, M. (2019). Palaeogeographic and chronostratigraphic reconstruction of the early human presence in Italy. Quaternary Newsletter 147: 39–41.
- Marra, F., Nomade, S., Pereira, A., Petronio, C., Salari, L., Sottili, G., Bahain, J.-J., Boschian, G., Di Stefano, G., Falguères, C., Florindo, F., Gaeta, M., Giaccio, B. (2018). A review of the geologic sections and the faunal assemblages of Aurelian Mammal Age of Latium (Italy) in the light of a new chronostratigraphic frameworkQuaternary Science Reviews 181: 173–199.

- Montes, L., Utrilla, P., Hedges, R. (2001). Le passage Paléolithique Moyen-Paléolithique Supérieur dans la Vallée de l'Ebre (Espagne), Datations radiométriques des grottes de Peña Miel et Gabasa. In: J Zilhao, T. Aubry y A. F. Caevalho (Eds.), Les premiers hommes modernes de la Péninsule Ibérique. Trabalhos de Arqueologia 17: 87–102.
- Nojiri-ko Group (1985). A Bone Scraper Excavated from Tategahana, Lake Nojiri, Central Japan. Quternary research 24: 111–124.
- Ono, A. (2006). Flacked bone tools and the Middle to Upper Paleolithic transition: a biref perspective. Archaeology, Ethnology and Anthropology of Eurasi 4: 38–47.
- Pereira, A., Sebastien Nomade, S., Moncel, M.-H. a, Voinchet, P., Bahain, J.-J., Biddittu, I., Falgueres, C., Giaccio, B., Manzi, G., Parenti, F., Scardia, G., Scao, V., Sottili, G., Vietti, A. (2018). Integrated geochronology of Acheulian sites from the southern Latium (central Italy): Insights on human-environment interaction and the technological innovations during the MIS 11-MIS 10 period. Quaternary Science Reviews 78: 112–129.
- Peresani, M., Vanharen, M., Quaggiotto, E., Queffelec, A., d'Errico, F. (2013). An ochred fossil marine shell from the Mousterian of FUmane Cae, Italy. PLOS ONE 8: e68572.
- Rendu, W., Kolobova, K.A., Shnaider, S., Augoyard, M., Gicqueau, A. (2020) ; Chez Pinaud (Commune de Jonzac, Charentes Maritimes), Rapport de fin d'opération annuelle 2020. University of Bordeaux I, Talence.
- Richter, D., Hublin, J.-J., Jaubert, J., McPherron, ShP., Soressi, M., Texier, J.-P. (2013). Thermoluminescence dates for the Middle Palaeolithic site of Chez-Pinaud Jonzac (France). Journal of Archaeological Science 40:1176–1185.
- Romandini, M., Cristiani, E. and Peresani, M. (2014). A retouched bone shaft from the Late Mousterian at Fumane cave (Italy), Technological, experimental and micro-wear analysis. Comptes Rendus Palevol 14: 63–72.
- Rosell, J., Blasco, R., Campeny, G., Díez, J.C., Alcalde, A.R., Menéndez, L., et al. (2011). Bone as technological raw material at the Gran Dolina site (Sierra de Atapuerca, Burgos, Spain). Journal of Human Evolution 6: 125-131.
- Rosell, J., Blasco, R., Fernández Peris, J., Carbonell, E., Barkai, R. and Gopher, A. (2015). Recycling bones in the Middle Pleistocene: Some reflections from Gran Dolina TD10-1 (Spain), Bolomor Cave (Spain) and Qesem Cave (Israel). Quaternary International 361: 297–312.
- Santucci, E., Marano, F., Cerilli, E., Fiore, I., Lemorini, C., Palombo, M.R., Anzidei, A.P., Bulgarelli, G.M. (2016). Palaeoloxodon exploitation at the Middle Pleistocene site of La Polledrara di Cecanibbio (Rome, Italy). Quaternary International 406: 169–182.
- Tartar, É., Costamagno, S. (2016). L'utilisation des matières osseuses au Moustérien. In: Turq, A., Faivre, J.-Ph., Maureille, B., Lahaye, Ch., Bayle, P. (eds), Néandertal à la loupe. Les Eyzies: Musée National de Préhistoire, pp. 89-97.
- Texier, P.-J. (1974). L'industrie moustérienne de l'abri Pié-Lombard (Tourettes-sur-Loup, Alpes-Maritimes). Bulletin de la Société préhistorique française 2: 429-448.
- Texier, P.-J. ; Renault-Miskosky, J., Desclaux,E., Lumley de, M.-A., Porraz, G., Tomasso A. (2011).L'abri Pié Lombard à Tourrettes-sur-Loup (Alpes-Maritimes) : Anciennes fouilles, nouvelles données. Bulletin du Musée d'Anthropologie préhistorique de Monaco 51: 19–49.
- Tromnau, G. (1983). Ein Mammutknochen-Faustkeil aus Rhede, Kreis Borken (Westfalen). Archäologisches Korrespondenzblatt, 13: 287–289.
- Villa, P., Boschian, G., Pollarolo, L., Saccà, D., Marra, F., Nomade, S., et al. (2021). Elephant bones for the Middle Pleistocene toolmaker. PLoS ONE 16 (doi.org/10.1371/journal.pone.0256090).
- Vincent, A. (1993). L'outillage osseux au Paléolithique moyen : une nouvelle approche. Thèse de Doctorat, Université Paris 10.
- Vuillemey, M. (1989). Les industries acheuléennes et moustériennes. In: Campy, M., Chaline, J., Vuillemey, M. (eds), La baume de Gigny. Gallia préhistoire supplément 27: 141-242.

- Zilhão, J., Cardoso, J.L., Pike, A.W.G., Weninger, B. (2011). Gruta Nova da Columbeira (Bombarral, Portugal): Site stratigraphy, age of the Mousterian sequence, and implications for the timing of Neanderthal extinction in Iberia. Quatär 58: 93-112.
