# Supplementary figures and images for: On the Quina side: A Neanderthal bone industry at Chez-Pinaud site, France
Source: PLoS One. 2023 Jun 14;18(6):e0284081. doi: 10.1371/journal.pone.0284081 (PMC10266661; doi:10.1371/journal.pone.0284081)

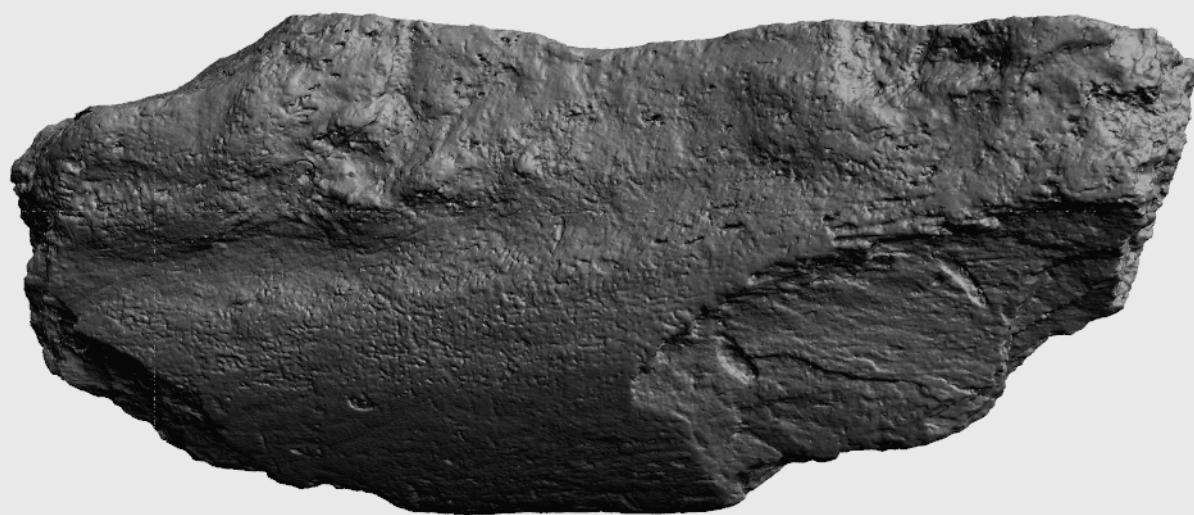

Fragment of retouched bone tool #CPN19-5, Chez-Pinaud at Jonzac.

Supplement: S1 File — (PDF) [file pone.0284081.s009.pdf]

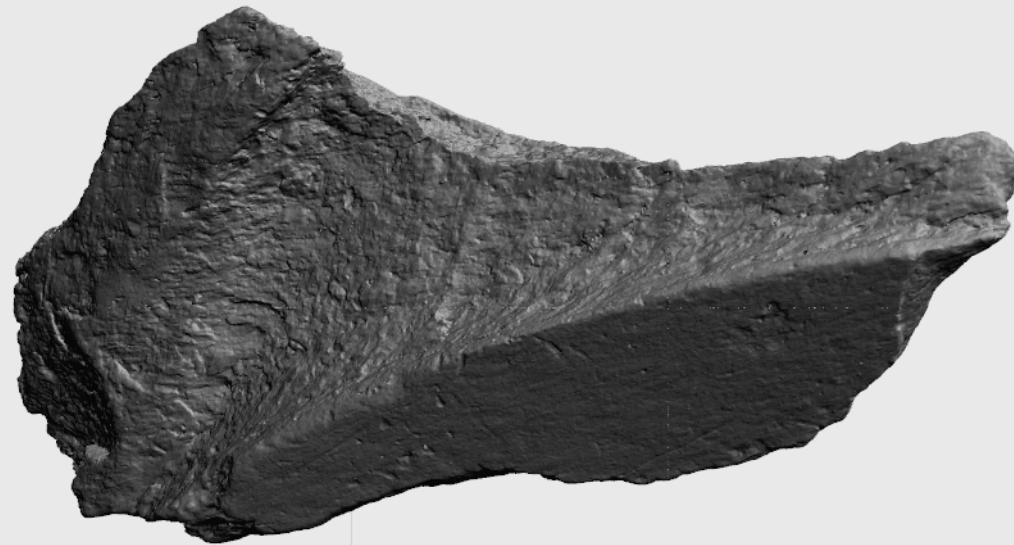

Fragment of retouched bone tool #CPN19-529, Chez-Pinaud at Jonzac

Supplement: S2 File — (PDF) [file pone.0284081.s010.pdf]

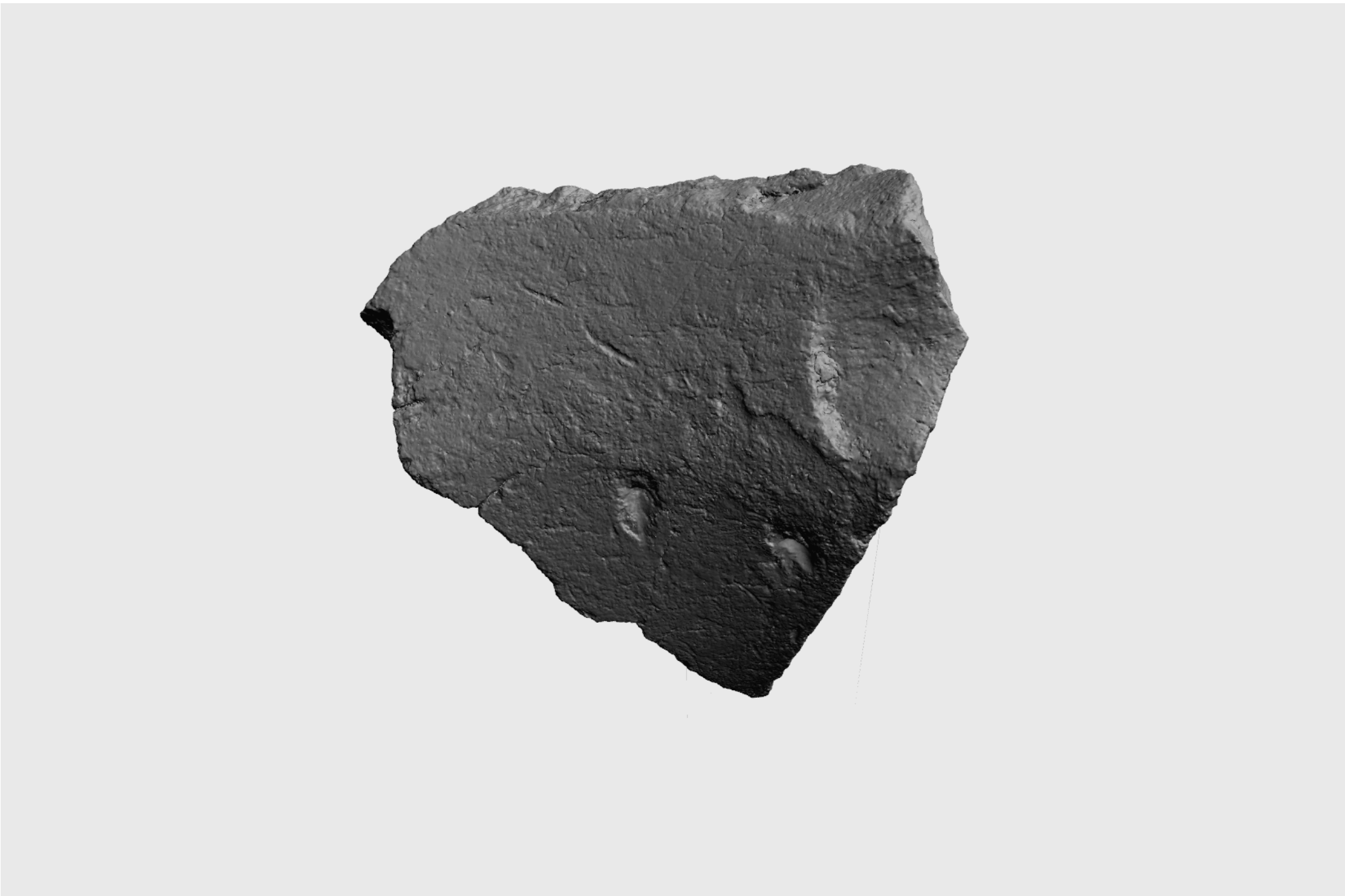

Beveled bone tool #CPN19-534, Chez-Pinaud at Jonzac

Supplement: S3 File — (PDF) [file pone.0284081.s011.pdf]

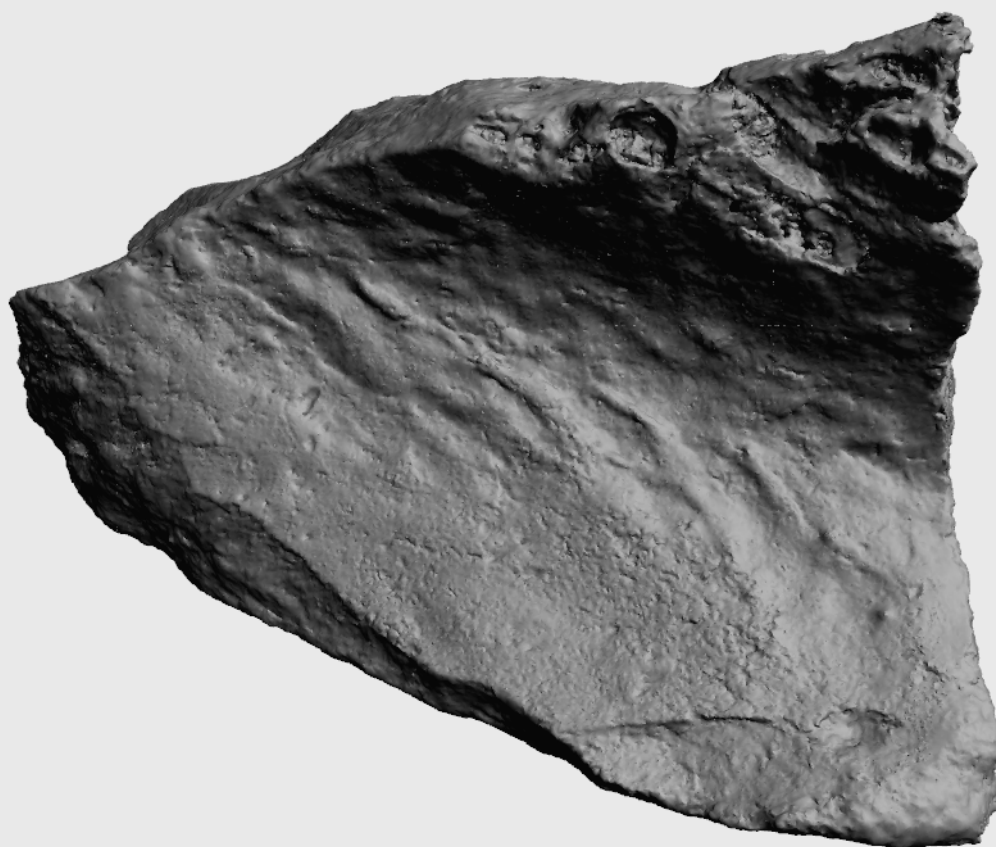

Beveled bone tool #CPN19-888, Chez-Pinaud at Jonzac

Supplement: S4 File — (PDF) [file pone.0284081.s012.pdf]

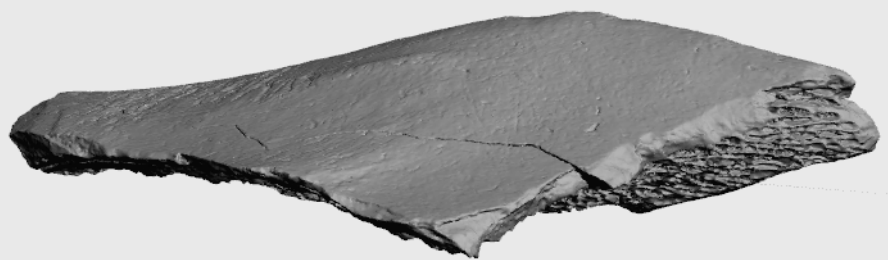

Beveled bone tool #CPN19-1014, Chez-Pinaud at Jonzac

Supplement: S5 File — (PDF) [file pone.0284081.s013.pdf]

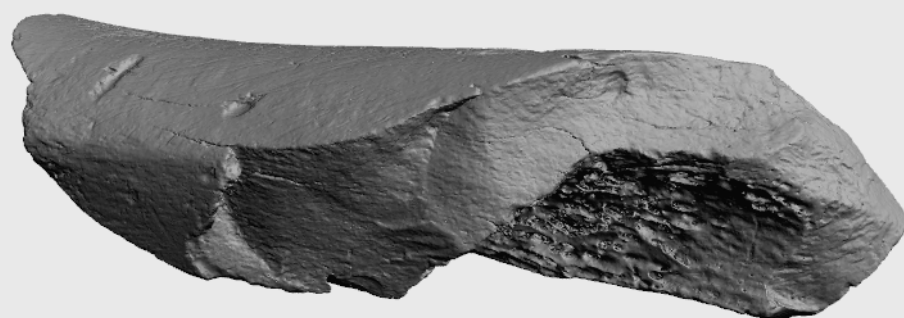

Bone retoucher #CPN19-2020, Chez-Pinaud at Jonzac

Supplement: S6 File — (PDF) [file pone.0284081.s014.pdf]

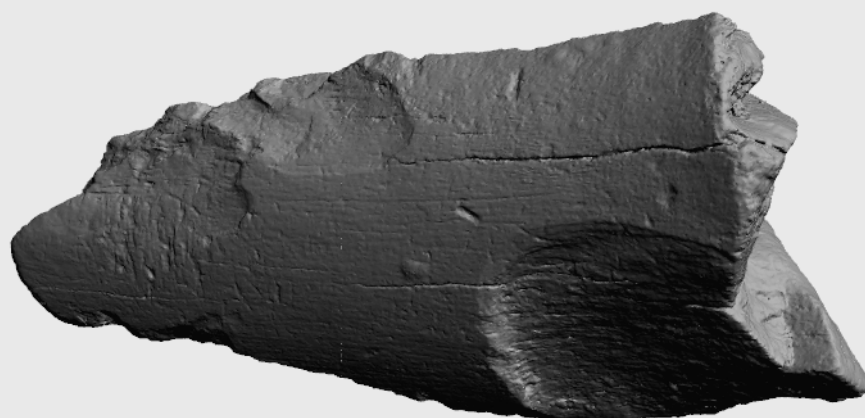

Retouched bone tool #CPN19-2132, Chez-Pinaud at Jonzac

Supplement: S7 File — (PDF) [file pone.0284081.s015.pdf]

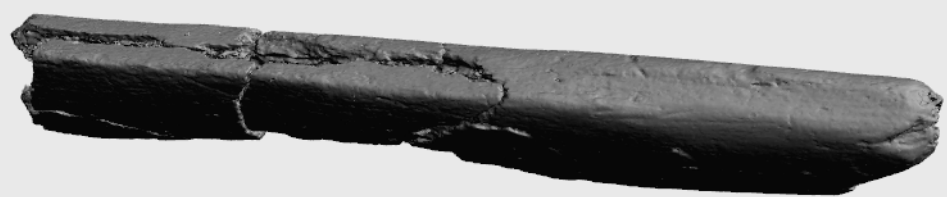

Smoothed-ended bone tool #CPN20-3581, Chez-Pinaud at Jonzac

Supplement: S8 File — (PDF) [file pone.0284081.s016.pdf]

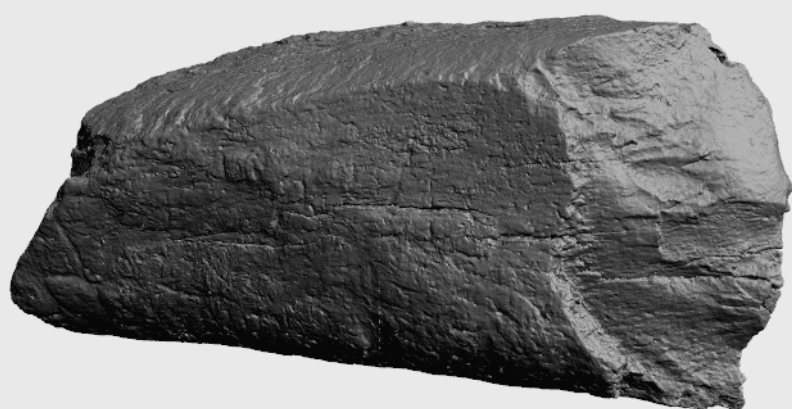

Beveled bone tool #CPN20-3609, Chez-Pinaud at Jonzac

Supplement: S9 File — (PDF) [file pone.0284081.s017.pdf]
