# Supplementary material for: On the Quina side: A Neanderthal bone industry at Chez-Pinaud site, France
Source: PLoS One. 2023 Jun 14;18(6):e0284081. doi: 10.1371/journal.pone.0284081 (PMC10266661; doi:10.1371/journal.pone.0284081)
Supplement: S1 Text — (PDF) [file pone.0284081.s018.pdf]

S1 Text. Bone retouchers

The bone retoucher is a common Middle Paleolithic bone tool (Armand and Delagnes 1998; Mallye et al. 2012; Jequier et al. 2012; Mozota Holgueras 2012; Blasco et al. 2013; Abrams et al. 2014; Deaujard et al. 2014; Rosell et al. 2015; Rougier et al. 2016; Doyon et al. 2018; Costamagno et al. 2018; Mateo-Lomba et al. 2019), which already exist in previous periods (Roberts and Parfitt 1999; Langlois 2004; Smith 2013; Julien et al 2015; Kolfschoten et al. 2015; Moigne et al. 2016) and last throughout the Upper Paleolithic (Patou-Mathis 2002; Castel et al 2003; Castel and Madelaine 2003; Rigaud 2007; Tartar 2009, 2012). Identified from its use-wear traces, it has benefited from functional analyses since the first specimens were discovered (Henri-Martin 1906). Most scholars agree that it is a light hammer for shaping lithic edges (Bonch-Osmolovskyi 1940; Semenov 1964; Feustel 1973; Rigaud 1977, 2007; Schelinskii 1983; Vincent 1988; Bourginon 2001; Schwab 2009; Mozota 2012; Mallye et al. 2012). But bone retouchers are seen as an undifferentiated unit, despite their morphometrical diversity, because of the scores apparent uniformity that does not allow to see significant differences between sites or chronocultures, apart from of the longitudinal orientation of the scores, specific to Upper Paleolithic material (Schwab 2002).

References

- Abrams, G., Bello, S.M., Modica, K. Di, Pirson, S. and Bonjean, D. (2014). When Neanderthals used cave bear (Ursus spelaeus) remains: Bone retouchers from unit 5 of Scladina cave (Belgium). Quaternary International 326–327: 274–287.
- Armand, D. and Delagnes, A. (1998). Les retouchoirs en os d'Artenac (couche 6c) : perspectives archéozoologiques, taphonomiques et expérimentales. In Brugal, J.-P., Meignen, L. and Patou-Mathis, M. (eds.), Économie Préhistorique : Les Comportements de Subsistance au Paléolithique, APDCA, Sofia Antipolis, pp. 205–214.
- Blasco, R., Rosell, J., Cuartero, F., Fernández Peris, J., Gopher, A. and Barkai, R. (2013). Using bones to shape stones: MIS 9 bone retouchers at both edges of the Mediterranean Sea. PLoS ONE 9: doi:10.1371/journal.pone.0076780
- Бонч-Осмоловский, Г.А. (1940). Палеолит Крыма. Грот Киик-Коба. Вып. 1. М.; Л. [Bonč-Osmolovskij G.A. (1940). Paleolit Kryma, Grot Kiik-Koba (Paleolithic of the Crimea. Kiik-Koba cave). Vyp. 1. M.; L].
- Bourguignon, L. (2001). Apports de l'expérimentation et de l'analyse techno-morpho-fonctionnelle à la reconnaissance du processus d'aménagement de la retouche Quina. In Bourguignon, L. and Frère-Sautot, M.C. (eds.), Préhistoire et Approche Expérimentale, Monique Mergoil, Montagnac, pp. 35–66.
- Castel, J.-C., Chauvière, F.-X. and Madelaine, S. (2003). Sur os et sur dents, les « retouchoirs » aurignaciens de la Ferrassie (Savignac-de-Miremont, Dordogne). Paléo 15: 29–50.
- Castel, J.-C. and Madelaine, S. (2003). Stigmates observés sur les dents de grands carnivores à l'Aurignacien, L'exemple de l'Abri de La Souquette à Sergeac (Dordogne, France). Paléo 15: 251–254.
- Costamagno, S., Bourguignon, L., Soulier, M.-C., Meignen, L., Beauval, C., Rendu, W., Mussini, C., Mann, A. and Maureille, B. (2018). Bone retouchers and site function in the Quina Mousterian: the case of Les Pradelles (Marillac-Le-Franc,France). In Huston, J.M., Garcia-

Monero, A., Noack, E.S., Turner, E., Villaluenga, A. and Gaudzinski-Windheuser, S. (eds.), The Origins of Bone Tool Technologies, RGZM, Tagungen, pp. 1–31.
- Daujeard, C., Moncel, M.-H., Fiore, I., Tagliacozzo, A., Bindon, P. and Raynal, J.-P. (2014). Middle Paleolithic bone retouchers in Southeastern France: Variability and functionality. Quaternary International 326–327: 492–518.
- Doyon, L., Li, Z., Li, H. and d'Errico, F. (2018). Discovery of circa 115,000-year-old bone retouchers at Lingjing, Henan, China. PLoS ONE 13: 1–16. doi:10.1371/journal.pone.0194318
- Feustel, R. (1973). Technik der Steinzeit, Archäolithikum-Mesoli-thikum, Hermann Böhlaus Nachfolger, Weimar.
- Henri-Martin, L. (1906). Maillets ou enclumes en os provenant de la couche moustérienne de la Quina (Charente). Bulletin de la Société préhistorique française 3: 155–162.
- Jéquier, C.A., Romandini, M. and Peresani, M. (2012). Les retouchoirs en matières dures animales : Une comparaison entre Moustérien final et Uluzzien. Comptes Rendus Palevol 11: 283–292.
- Julien, M.-A., Hardy B., Stahlschmidt, M.C., Urban, B., Serangeli, J., Conard, N.J. (2015). Characterizing the Lower Paleolithic bone industry from Schöningen 12 II: A multi-proxy study. Journal of Human Evolution 89: 264–286.
- Kolfschoten, T.V., Parfitt, S.A., Serangeli, J. and Bello, S.M. (2015). Lower Paleolithic bone tools from the 'Spear Horizon' at Schöningen (Germany). Journal of Human Evolution 89: 226–263.
- Langlois, A. (2004). Au sujet du Cheval de La Micoque (Dordogne) et des comportements humains de subsistance au Pléistocène moyen dans le Nord-Est de l'Aquitaine. Thèse de Doctorat, Université de Bordeaux I.
- Mallye, J.B., Thiébaut, C., Mourre, V., Costamagno, S., Claud, É. and Weisbecker, P. (2012). The Mousterian bone retouchers of Noisetier Cave: Experimentation and identification of marks. Journal of Archaeological Science 39: 1131–1142.
- Mateo-Lomba, P., Rivals, F., Blasco, R. and Rosell, J. (2019). The use of bones as retouchers at Unit III of Teixoneres Cave (MIS 3; Moià, Barcelona, Spain). Journal of Archaeological Science Report 27: 101980. doi:10.1016/j.jasrep.2019.101980
- Moigne, A.-M., Valensi, P., Auguste, P., García-Solano, R., Tuffreau, A., Lamotte, A., Barroso, C., Moncel, M.-H. (2016). Bone retouchers from Lower Palaeolithic sites: Terra Amata, Orgnac 3, Cagny-l'Epinette and Cueva del Angel. Quaternary International 409: 195–212.
- Mozota Holgueras, M.M. (2012). El hueso como materia prima: El utillaje óseo del final del Musteriense en el sector central del norte de la Península Ibérica. Tesis Doctoral, Universidad de Cantabria.
- Patou-Mathis, M. (2002). Fiches Typologiques de l'Industrie Osseuse Préhistorique, Cahier X : Retouchoirs, Compresseurs, Percuteurs, Os à Impressions et Éraillures, Publications de l'Université de Provence, Aix-en-Provence.
- Rigaud, A. (1977). Analyse typologique et technologique des grattoirs magdaléniens de La Garenne à Saint-Marcel (Indre). Gallia Préhistoire 20: 1–43.
- Rigaud, A. (2007). Retouchoirs sur éclats diaphysaires ou « affûtoirs » de Labastide (Hautes-Pyrénées), Du Barbarisme scientifique à la rigueur artisanale au travers de l'expérimentation. Archéologie des Pyrénées Occidentales et des Landes 26: 193–200.
- Roberts, M. and Parfitt, S. (1999). A Middle Pleistocene Hominid Site at Eartham Quarry, Boxgrove, West Sussex. English Heritage, London.
- Rosell, J., Blasco, R., Fernández Peris, J., Carbonell, E., Barkai, R. and Gopher, A. (2015). Recycling bones in the Middle Pleistocene: Some reflections from Gran Dolina TD10-1 (Spain), Bolomor Cave (Spain) and Qesem Cave (Israel). Quaternary International 361: 297–312.
- Rougier, H., Crevecoeur, I., Beauval, C., Posth, C., Flas, D., Wißing, C., Furtwängler, A., Germonpré, M., Gómez-Olivencia, A., Semal, P., Van Der Plicht, J., Bocherens, H. and Krause,

J. (2016). Neandertal cannibalism and Neandertal bones used as tools in Northern Europe. Science Report 6: 1–11.
- Щелинский, В.Е. (1983). К изучению техники, технологии изготовления и функций орудий мустьерской эпохи. Рогачёв АН (ред), Технология производства в эпоху палеолита, Наука, Ленинград, 72–133. [Ŝelinskij, V.E. (1983.) K izučeniû tehniki, tehnologii izgotovleniâ i funkcij orudij must′erskoj èpohi (Towards the study of technic, manufacturing technology and functions of tools of the Mousterian epoch). In Rogachjov, A.N. (ed.), Tehnologiâ proizvodstva v èpohu paleolita (Production technology in the Paleolithic era), Nauka, Leningrad, 72–133.]
- Schwab, C. (2002). Fiche éclats diaphysaires du Paléolithique moyen et supérieur : la Grotte d’Isturitz (Pyrénées-Atlantiques). In Patou-Mathis, M. (ed.), Fiches Typologiques de l'Industrie Osseuse Préhistorique, Cahier X : Retouchoirs, compresseurs, percuteurs…Os à impressions et éraillures, SPF, Paris, pp. 59–73.
- Schwab, C. (2009). Les “os à impressions et à éraillures”: premiers résultats expérimentaux. Antiquités Nationales 40: 29–37.
- Semenov, S.A. (1964). Prehistoric Technology, an Experimental Study of the Oldest Tools and Artefacts from Traces of Manufacture and Wear, Cory, Adams & Mackay, London.
- Smith, G.M. (2013). Taphonomic resolution and hominin subsistence behaviour in the Lower Palaeolithic: differing data scales and interpretive frameworks at Boxgrove and Swanscombe (UK). Journal of Archaeological Science 40: 3754–3767.
- Tartar, É. (2009). De l’os à l’outil, caractérisation technique, économique et sociale de l’utilisation de l’os à l’Aurignacien ancien, Etude de trois sites : l’Abri Castanet (secteur nord et sud), Brassempouy (Grotte des Hyènes et Abri Dubalen) et Gatzarria. Thèse de Doctorat, Université Paris I.
- Tartar, É. (2012). Réflexion autour de la fonction des retouchoirs en os de l’Aurignacien ancien. Bulletin de la Société préhistorique française 109: 69–83.
- Vincent, A. (1988). L’os comme artefact au Paléolithique moyen : principes d’étude et premiers résultats. In Otte, M. (ed.), L'Homme de Néandertal, Vol. 4: La Technique. ERAUL, Liège, pp. 185–196.
