# Supplementary material for: On the Quina side: A Neanderthal bone industry at Chez-Pinaud site, France
Source: PLoS One. 2023 Jun 14;18(6):e0284081. doi: 10.1371/journal.pone.0284081 (PMC10266661; doi:10.1371/journal.pone.0284081)
Supplement: S2 Text — (PDF) [file pone.0284081.s019.pdf]

S2 Text. Beveled tools

Beveled tools, mainly made from antler, are frequently identified in Upper Paleolithic contexts (Deffarges et al. 1974; Provenzano 1984). In later periods, Mesolithic and Neolithic, bone specimens are more common (Camps-Fabrer et al. 1998; Maigrot 2003). In techno-traceological studies, chisels (for cutting) and wedges (for splitting) are often grouped together (Provenzano 1998) because they share the same characteristics: a cutting edge at one end marked by crushing sometimes associated with a blunt area, chips or small removals, and striations oriented along the main axis of the tool. The force imparted to set them in motion can be transmitted directly by the arm, or indirectly with a hammer. In the latter case, the tools are characterized by a striking surface located on the end opposite to the bevel and materialized by a crushing area surrounded by macro to micro removals. However, the absence of a striking surface does not necessarily indicate that a hammer has not been used. This occurs when the tools are hafted, such as the bone bevels from the Swiss Final Neolithic (Voruz 1984). If the handle is not preserved, it can be revealed by: (1) the proximal end shaping (Sidéra 1989), (2) a glossing/blunting of the proximal tool edges (Maigrot 1997), a clear limit of the distal use-wear traces, or a clear change of the bone material coloration (Maigrot 2003). Repeated hammering of the proximal end can also lead to fatigue fractures. These use-fractures are reportedly quite common on bone beveled tools (Tartar 2012; Maigrot et al. 2013).

References

- Camps-Fabrer, H, Cattelain, P., Choï, S.-Y., David, E., Pascual-Benito, J.-L., Provenzano, N. and Ramseyer, D. (1998). Fiches Typologiques de l'Industrie de l'Os Préhistorique, Cahier VIII : Biseaux et Tranchants, CEDAR, Treignes.
- Deffarges, R., Laurent, P. and de Sonneville-Bordes, D. (1974). Ciseaux ou lissoirs magdaléniens. Bulletin de la Société préhistorique française 71: 85–96.
- Maigrot, Y. (1997). Tracéologie des outils tranchants en os des Ve et IVe millénaires av. J.-C. en Bassin parisien, Essai méthodologique et application. Bulletin de la Société préhistorique française 94: 198–216.
- Maigrot, Y. (2003). Étude technologique et fonctionnelle de l'outillage en matières dures animales, La station 4 de Chalain (Néolithique final, Jura, France). Thèse de Doctorat, Université Paris I.
- Maigrot, Y., Clemente Conte, I., Gyria, E., Lozovskaya, O. and Lozovski, V. (2013). Analyse fonctionnelle des outils biseautés à 45° de Zamostje 2. In Lozovski, V.M., Lozovskaya, O.V. and Clemente Conte, I. (eds.), Zamostje 2, Lake Settlement of the Mesolithic and Neolithic Fisherman in Upper Volga Region, Russian Academy of Science, St-Petersburg, pp. 120–140
- Provenzano, N. (1984). Coins et ciseaux au Paléolithique supérieur. Mémoire de Master, Université de Provence.
- Provenzano, N. (1998). Fiche générale des objets à biseau distal. In Camps-Fabrer, H. (eds.), Fiches typologiques de l'industrie osseuse préhistorique, Cahier VIII: biseaux et tranchants, Commission de nomenclature sur l'industrie osseuse préhistorique, CEDARC, Treignes, pp. 5-16.
- Sidéra, I. (1989). Un complément des données sur les sociétés rubanées, l'industrie osseuse de Cuiry-lès-Chaudardes. BAR, Oxford.
- Tartar, É. (2012). The recognition of a new type of bone tools in Early Aurignacian assemblages: Implications for understanding the appearance of osseous technology in Europe. Journal of Archaeological Science 39: 2348–2360.

- Voruz, J.-L. (1984). Outillage Osseux et Dynamisme Industriel dans le Néolithique Jurassien, Bibliothèque Historique Vaudoise, Lausanne.
