# Supplementary material for: On the Quina side: A Neanderthal bone industry at Chez-Pinaud site, France
Source: PLoS One. 2023 Jun 14;18(6):e0284081. doi: 10.1371/journal.pone.0284081 (PMC10266661; doi:10.1371/journal.pone.0284081)
Supplement: S3 Text — (PDF) [file pone.0284081.s020.pdf]

S3 Text. Retouched tools

The identification of an intentional retouch on bone is often put forward when all other causes that could lead to removals have been ruled out (Vincent 1993). The archaeological context allows the diagnosis to be based with greater confidence (Inizan et al. 1995). At Chez-Pinaud, the absence of carnivore consumption traces (chewing, grooves, pits, punctures, and digestion blunts; Stucliffe 1973; Haynes 1983; Campas and Beauval 2008; Fourvel 2012), dispels the first source of possible confusion regarding the notches origin. The likelihood that the bone blanks were transformed by humans for technical purposes is here increased by their tool status: most were used as retouchers. Among the technical causes, bone fracturing by percussion during butchery processes also lead to the formation of removals (medullary or cortical side). In this case, the latter are related to the adjacent fracture, and sometimes to traces of the anvil's counter-strike on the opposite face, all resulting from a single event, generally a violent blow given perpendicular to the bone (Capaldo and Blumenschine 1994; Pickering and Egeland 2006).

Although there are few references about the intentional retouch of bone, positive clues of identification can nevertheless be used. Lithic knapping provides keys to understanding that can partially be transferred to bone insofar as the latter behaves like a conchoidal fracture material. However, bone is also a fibrous material whose mechanical properties vary according to the stress direction. Therefore, it responds to knapping in a more complex way than flint, due to its anisotropic structure. Nevertheless, common regularities and characteristics can be observed, implying the same reasoning in their analysis. The organization of the removals is the main criterion to consider for identifying an intentional knapping (Lyman 1984; Vincent 1993; Inizan et al. 1995).

References

- Campas, E. and Beauval, C. (2008). Consommation osseuse des carnivores : résultats de l'étude de l'exploitation des carcasses de bœufs (Bos taurus) par des loups captifs. Annales de Paléontologie 94: 167–186.
- Capaldo, S.D. and Blumenschine, R.J. (1994). A quantitative diagnosis of notches made by hammerstone percussion and carnivore gnawing on bovid long bones. American Antiquity 59: 724–748.
- Fourvel, J.-B. (2012). Hyénidés modernes et fossiles d'Europe et d'Afrique : Taphonomie comparée de leurs assemblages osseux. Thèse de Doctorat, Université de Toulouse.
- Haynes, G. (1983). Frequencies of spiral and green-bone fractures on ungulate limb bones in modern surface assemblages. American Antiquity 48: 102–114.
- Inizan, M.-L., Reduron-Ballinger, M., Roche, H. and Tixier, J. (1995). Préhistoire de la pierre taillée, IV - Technologie de la pierre taillée. Cercle de recherches et d'études préhistoriques, Meudon.
- Lyman, R.L. (1984). Broken bones, bone expediency tools, and bone pseudotools: Lessons from the Blast zone around Mount St. Helens, Washington. American Antiquity 49: 315–333.
- Pickering, T.R. and Egeland, C.P. (2006). Experimental patterns of hammerstone percussion damage on bone: Implications of inferences of carcass processing by human. Journal of Archaeological Science 33: 459–469.

- Sutcliffe, A.J. (1973). Similarity of bones and antlers gnawed by deer to Human artefacts. Nature 246: 428–430.
- Vincent, A. (1993). L’outillage osseux au Paléolithique moyen : une nouvelle approche. Thèse de Doctorat, Université Paris 10.
