# Supplementary material for: On the Quina side: A Neanderthal bone industry at Chez-Pinaud site, France
Source: PLoS One. 2023 Jun 14;18(6):e0284081. doi: 10.1371/journal.pone.0284081 (PMC10266661; doi:10.1371/journal.pone.0284081)
Supplement: S4 Text — (PDF) [file pone.0284081.s021.pdf]

S4 Text. Smooth-ended tools

Blunts are among the most common bone alterations induced by a variety of post-depositional processes (Sutcliffe 1973; Shipman and Rose 1988; Olsen 1989; Villa and d'Errico 2001; Fernández-Jalvo and Andrews 2013). The main criteria for differentiating a technical blunt from a taphonomic one is its extension, location, orientation, its transition with adjacent surfaces, and the combination of these different features. Natural erosion or dissolution tends to cover most, if not all, of the bone surface, whereas a blunt from use is restricted to the surface or edge of the active area. Hafting or gripping blunts may be more extensive, but remain related to a particular tool area. Blunt from friction into the sediments is mainly superficial and does not affect the concavities. The tool effectiveness depends on the control of its working angle. Maintaining this angle induces a regularity of the use-wear characteristics. The profile of the active edge rounding (seen in cross section) thus depends on the way the tool is used but also on the worked material physical properties, depending on whether the latter is organic or mineral, dry, fresh or wet, compact or granular, fibrous or not, etc. The maximum development of the bluntness is fixed by the minimum sharpness necessary to obtain the expected results on the worked material.

References

- Fernández-Jalvo, Y. and Andrews, P. (2016). Atlas of Taphonomic Identifications, Springer Dordrecht, New York.
- Olsen, S.L. (1989). On distinguishing natural from cultural damage on archaeological antler. Journal of Archaeological Science 16: 125–135.
- Shipman, P. and Rose, J.J (1988). Bone tools: An experimental approach. In Olsen, S. (ed.), Scanning Electron Microscopy in Archaeology, BAR International, Oxford, pp. 303–336.
- Sutcliffe, A.J. (1973). Similarity of bones and antlers gnawed by deer to Human artefacts. Nature 246: 428–430.
- Villa, P. and d'Errico, F. (2001). Bone and ivory points in the Lower and Middle Paleolithic of Europe. Journal of Human Evolution 41: 69–112.
