# Supplementary material for: On the Quina side: A Neanderthal bone industry at Chez-Pinaud site, France
Source: PLoS One. 2023 Jun 14;18(6):e0284081. doi: 10.1371/journal.pone.0284081 (PMC10266661; doi:10.1371/journal.pone.0284081)
Supplement: Attachment — Submitted filename: Response to Reviewers.docx [file pone.0284081.s023.docx]

Response to Reviewers

**Reviewer 1**

Lines 67-68: Grammar here could be improved. Do you mean ‘To date, the Chagyrskaya bone tools provide the only example of a Neanderthal bone 68 industry *for which* the authorship of AMH cannot be considered’?

Done

Line 67-69: I am confused by this statement. If Chagyrskaya is the *only* example of a Neanderthal bone industry, how can you state just after that it is *not an isolated* case?

“The bone tools from Chagyrskaya are not an isolated case” have been replaced by "Bone tools have already been reported in Neanderthal sites but most of the time as isolated finds.".

Lines 80-81: This sentence is unclear. What does ‘others’ refer to? Other discoveries? Other industries? Please rephrase.

The sentence has been replaced by " These discoveries, most of them recently investigated, allow us to reconsider **earlier ones** which were potentially too readily dismissed"

Lines 283-284: rather than ‘close’ striking angle I would say ‘acute’.

Done

Line 383: replace ‘Bos/bison’ with ‘Bos/Bison’ (capitalised words and italic).

We capitalized and italicized Bos (taxa) but not bison as it is the common vernacular name.

Line 415: ‘The US22’. Remove ‘The’.

Done

Line 467: Adjust the grammar here. Change the sentence to either ‘the mode of use of *a* bone retoucher […]’ or ‘the mode of use of bone *retouchers* can also be revealed by *their* internal damage.

Done

Line 468: replace ‘reciprocal effect of one on the other’ with ‘reciprocal effect on one another’

Done

Line 564: replace ‘in the main axis’ with ‘along its main axis’.

Done

Line 596: replace 2 and 4 with ‘two’ and ‘four’.

Done

Line 710: replace ‘done by percussion’ with ‘produced by, resulting from, achieved through…’ percussion.

Done

Line 853: correct spelling is ‘De Nadale’.

Done
